# Case Report: Successful treatment of refractory synovitis, acne, pustulosis, hyperostosis, and osteitis syndrome and palmoplantar pustulosis with ustekinumab

**DOI:** 10.3389/fimmu.2025.1628279

**Published:** 2025-07-10

**Authors:** Luxia Chen, Qichang Liang, Sijia Chen, Hao Cheng

**Affiliations:** ^1^ Department of Dermatology and Venereology, Sir Run Run Shaw Hospital, Zhejiang University School of Medicine, Hangzhou, China; ^2^ Department of Dermatology, Children’s Hospital, Zhejiang University School of Medicine, Hangzhou, China

**Keywords:** SAPHO syndrome, palmoplantar pustulosis, anti-IL-12/23 antibody, ustekinumab, immune-mediated inflammatory diseases

## Abstract

Synovitis, acne, pustulosis, hyperostosis, and osteitis (SAPHO) syndrome is a rare chronic inflammatory disease mainly manifested as skin and osteoarticular lesions. We describe a male patient with SAPHO syndrome who exhibited primary palmoplantar pustulosis (PPP). Notably, his condition worsened during treatment with adalimumab and other immunosuppressants. After switching to ustekinumab, the patient obtained significant improvement in both skin lesions and osteoarticular pain. These findings suggest that ustekinumab may represent an appropriate option for patients with refractory SAPHO syndrome.

## Introduction

The synovitis–acne–pustulosis–hyperostosis–osteitis (SAPHO) syndrome represents a clinically heterogeneous disorder, characterized by inflammatory osteoarticular lesions accompanied by diverse dermatological manifestations. These skin conditions range from acne, psoriasis, and palmoplantar pustulosis to hidradenitis suppurativa, pyoderma gangrenosum, and Sweet syndrome, reflecting the syndrome’s broad phenotypic spectrum (1).

While the pathogenesis of SAPHO syndrome has not been fully elucidated, current evidence suggests that genetic predisposition, dysregulated inflammatory responses, and microorganisms, for example, propionibacterium acnes, may contribute to disease development (2, 3). Notably, specific genetic variants, including mutations in the Proline-serine-threonine phosphatase interacting protein 2 (PSTPIP2) and Nucleotide-binding oligomerization domain-containing protein 2 (NOD2) genes, have been implicated in the onset of SAPHO syndrome; however, these associations require further validation in larger studies (4). Additionally, multiple inflammatory factors, including Tumor Necrosis Factor (TNF)-α, Interleukin (IL)-1β, IL-8, IL-17, and IL-18, have been implicated in SAPHO syndrome pathogenesis, contributing to the complexity of treatment (5).

Current therapeutic strategies for SAPHO syndrome increasingly incorporate biologic agents, which have demonstrated efficacy in ameliorating both osteoarticular pain and cutaneous manifestations. The central role of IL-1 and TNF-α in the syndrome’s inflammatory process has been well-established, with clinical trials of inhibitors like anakinra and infliximab demonstrating benefits in reducing symptoms and enhancing patient quality of life (3).

Nevertheless, some patients might still develop severe rashes during treatment with TNF-α inhibitors, which led physicians to select appropriate therapeutic options to treat SAPHO syndrome. The management of SAPHO syndrome remains clinically challenging, particularly in target selection. Recent studies have concentrated on identifying novel biomarkers and therapies that influence the disease’s progression. For instance, the IL-23/Th17 pathway has emerged as a promising therapeutic target in SAPHO syndrome (6).

Ustekinumab is a fully human IgG1/κ monoclonal antibody that specifically binds to the shared p40 subunit of IL-12 and IL-23, thereby blocking their downstream signaling pathways. This mechanism underlines its therapeutic potential in Th1- and Th17-mediated inflammatory disorders. We present a case of refractory SAPHO syndrome with concomitant palmoplantar pustulosis (PPP) that demonstrated significant improvement in both cutaneous and osteoarticular manifestations following ustekinumab therapy, with no observed adverse effects.

## Case description

A 38-year-old male patient presented with erythema and pustules on the palmoplantar area without obvious causes for 1 year and 2 months, along with pain in the sternoclavicular joints starting from 1 month before the skin lesion occurred. Medical history revealed that he was diagnosed with palmoplantar pustulosis (PPP) at a local clinic 1 year earlier and was given oral glucocorticoids (precise dosage unclear) for 3 days, and his lesions were not in remission. Laboratory tests showed elevated C-reactive protein (CRP) and erythrocyte sedimentation rate (ESR). The patient was prescribed Acitretin Capsules (10 mg, once every night), Compound Aminopeptide Tablets (three tablets, three times a day), and Compound Glycyrrhizin Tablets (25 mg, three times a day), along with topical Compound Miconazole Cream, Compound Tretinoin Cream, and Compound Clotrimazole Cream. However, the treatment proved to be ineffective. 6 months earlier, the patient was treated with adalimumab for 2 months. After the first four doses, there was an improvement with no new pustules and reduced sternum pain. However, after the fifth injection, the patient developed a facial infection and new pustules on the trunk. Treatment was then switched to acitretin and Thunder God Vine Glycosides, but during the process of reducing the dosage, the patient experienced recurrent skin rashes on the trunk and sternum pain. 3 months before admission, the patient got low back pain, which worsened significantly after 2 months, with pain upon walking and turning. MRI results in a local clinic indicated L3 and L4 compression fractures, L3, L4, and L5 vertebral bone marrow edema, and L5-S1 disc bulging.

**Figure 1:**
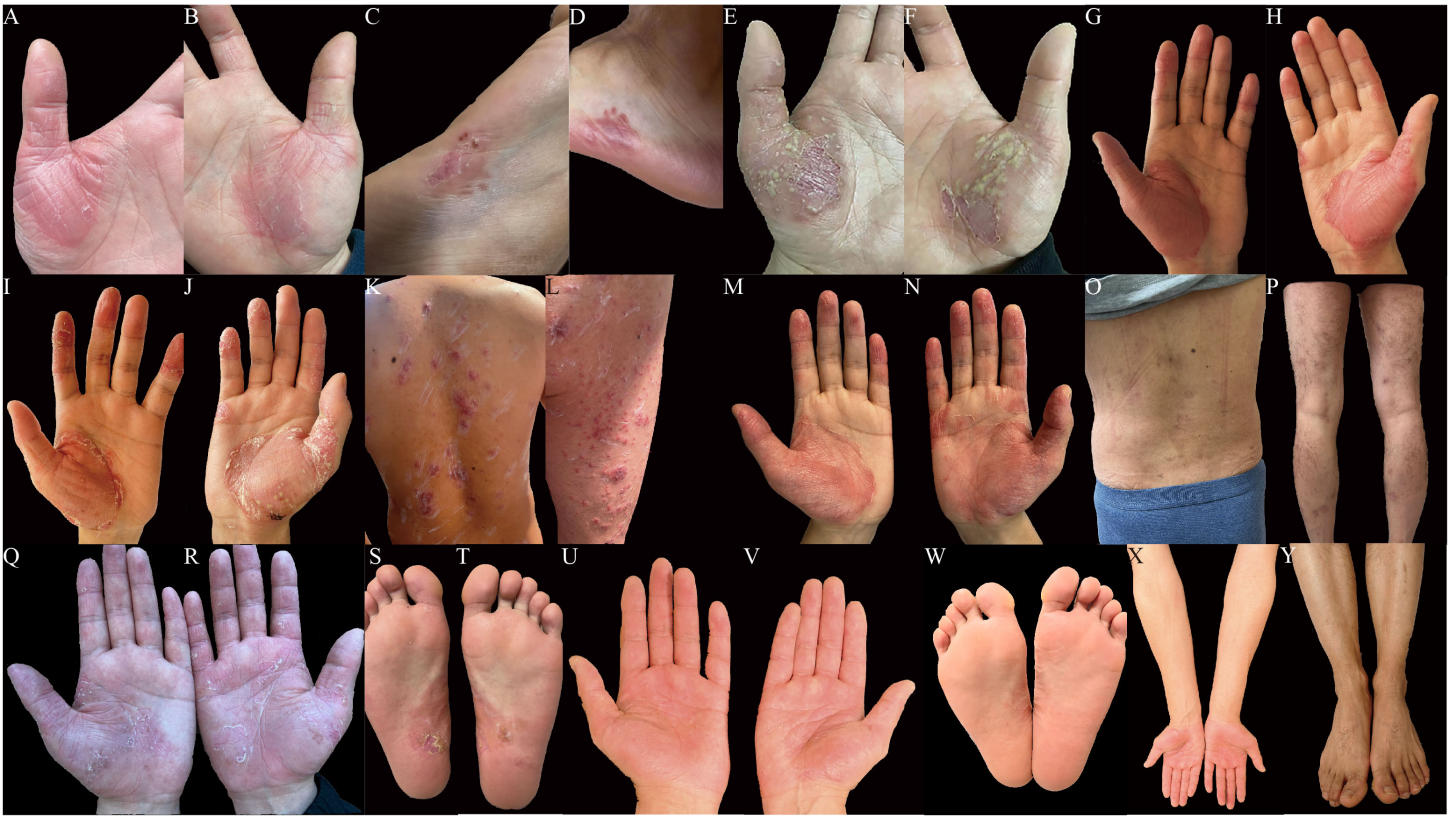
**(A-Y)** Cutaneous manifestations. Erythema and desquamation on the palms **(A, B)** and the lateral side of the feet **(C, D)** in the beginning. Severe pustules and crusts on the palms **(E, F)** before the administration of adalimumab. No pustules and enlarged erythema on the palms **(G, H)** after 4-dose administration of adalimumab. Newly erupted pustules and crusts on the palms **(I, J)**, multiple erythematous papules and pustules on the trunk **(K)** and lower limbs **(L)** after 5-dose administration of adalimumab. No pustules and improved erythema on the palms **(M, N)** after 1-dose administration of ustekinumab. Disappeared erythematous and scattered pigmentation on the trunk **(O)** and legs **(P)** after 3-dose administration of ustekinumab. Scattered desquamation and dark red patches on palms **(Q, R)** and soles **(S, T)** after 4-dose administration of ustekinumab (90mg) and 2-dose administration of ustekinumab (45mg). Slight desquamation, disappeared pustules on palms **(U, V)** and soles **(W)**, recovered skin on extremities **(X, Y)** after 2.5 years of ustekinumab treatment: 4 doses of ustekinumab (90mg) and 6 doses of ustekinumab (45 mg) each 12 weeks (photos taken recently).

At the time of admission, the patient was still suffering from sternum pain and low back pain, widespread erythema with scales, and large erythematous patches on the palms and soles. The progression of skin rashes prior to admission is demonstrated in **Figures 1A–L**. The patient denied any symptoms including chest tightness, chills, fever, or abdominal discomfort, and had no history of gastrointestinal symptoms or psoriasis. For further evaluation and treatment, the patient was admitted with a diagnosis of palmoplantar pustulosis and suspected SAPHO syndrome.

Dermatological examination revealed erythematous patches with well-defined boundaries on the palms and soles. Several scattered pustules were seen at the boundary. Large areas of scaling were observed on the thenar eminence and palmar surface of the fingers. Multiple erythema and scaling were present on the trunk, and scattered red maculopapular rashes were seen on the limbs. Physical examination revealed tenderness over the sternum and lumbar back region, with pain aggravated during standing, walking and turning over.

Laboratory tests revealed the following: ESR, 61 mm/h [normal range <15 mm/h]; CRP, 27.9 mg/L [normal range <6 mg/L]; human leukocyte antigen-B27, negative; and anti-nuclear antibodies, negative.

**Figure 2:**
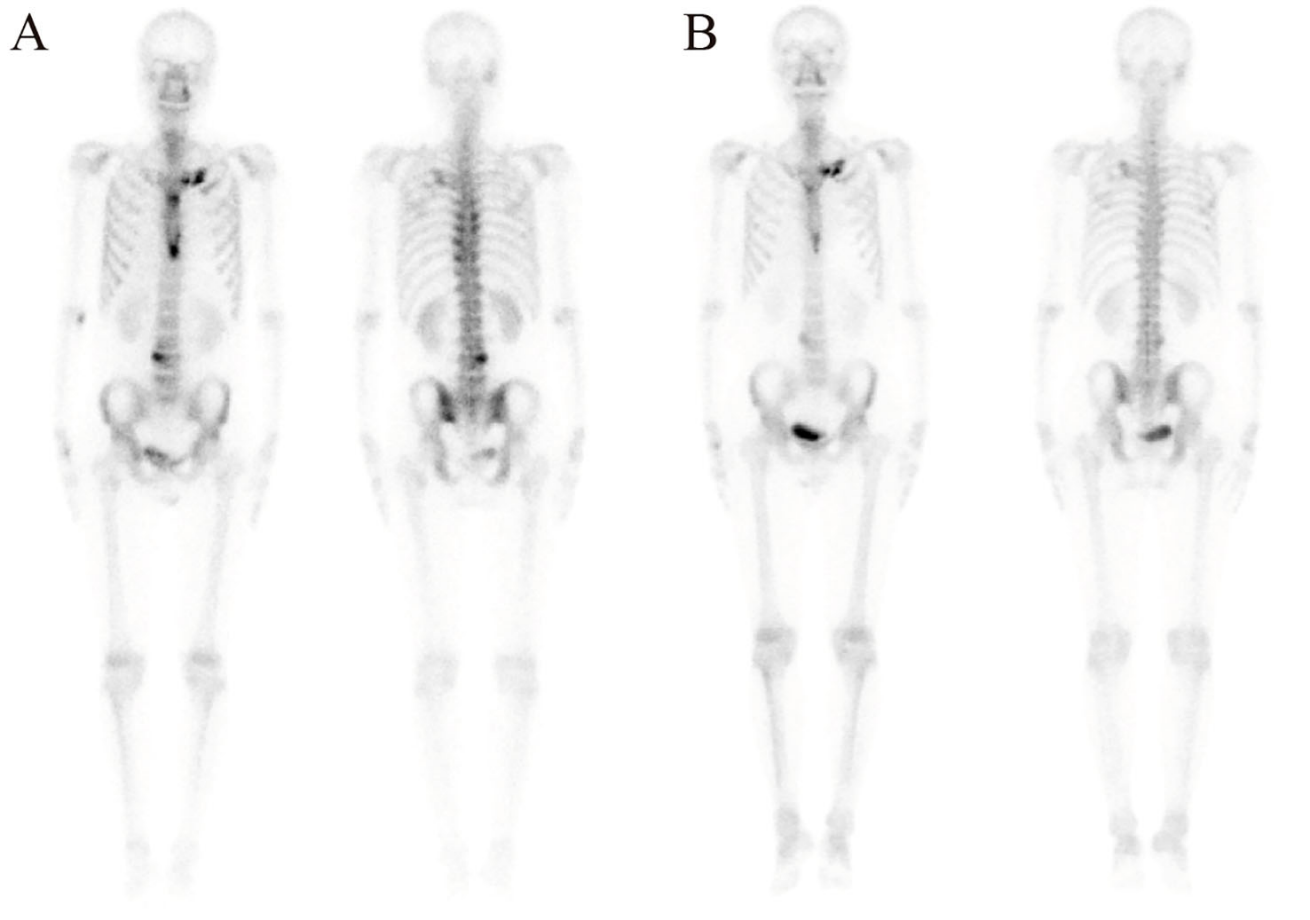
Inflammatory changes and the “bull’s head” sign in 99mTc-methylene diphosphonate whole-body bone scintigraphy before **(A)** and after **(B)** treatment with ustekinumab for 10 months.

**Figure 3:**
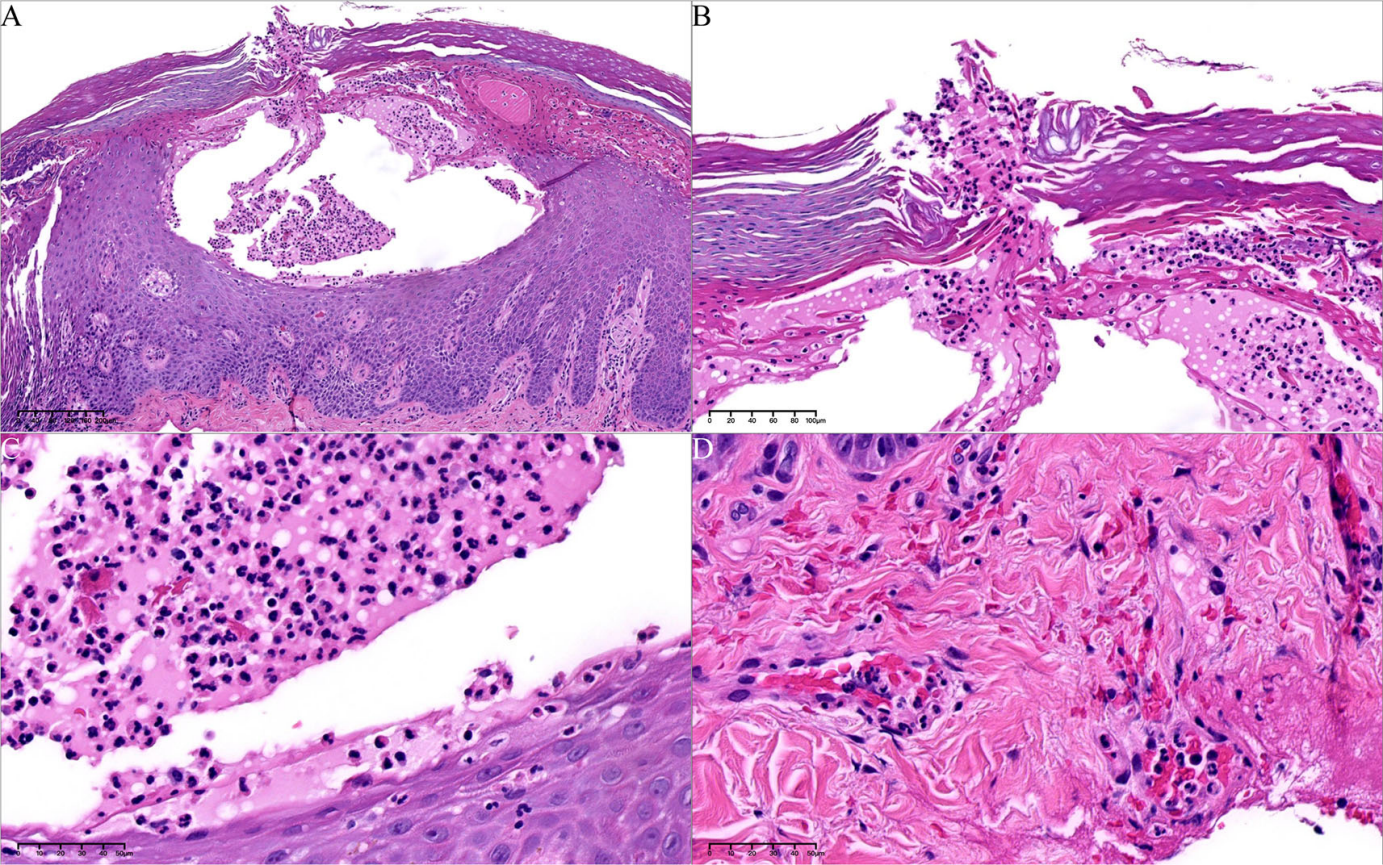
Histopathology of palm pustules. **(A)** Pustules in epidermis, with hemorrhagic and necrotic crusts (hematoxylin–eosin [HE], 80*). **(B)** The epidermal vesicle contains mononuclear cells and neutrophils (arrow) (HE, 100*). **(C)** Microabscesses are evident at the edges of the vesicle (HE, 400*). **(D)** Perivascular infiltration of neutrophils and lymphoid cells in dermis (HE, 400*).

99mTc-methylene diphosphonate whole-body bone scintigraphy revealed inflammatory changes in the sternal angle, left sternoclavicular joints, the left 1st and 2nd costosternal junctions, the 7th to 10th thoracic vertebrae costovertebral joints, and the 4th lumbar vertebra. The pathological uptake of the anterior chest wall presented the “incomplete” “bull’s head” sign (**Figure 2A**). Based on the comprehensive clinical assessment, the diagnosis of SAPHO syndrome was confirmed. Skin biopsy of palm lesion indicated (**Figure 3**): hyperkeratosis, parakeratosis, with hemorrhagic necrotic crusts observed. Pustules with neutrophil aggregation are present in the stratum corneum and epidermis. In the superficial dermis, there is vascular dilation and congestion, with erythrocyte extravasation. Around the vessels, there is mild to moderate infiltration of neutrophils and lymphoid cells. The pathological diagnosis of palmoplantar pustulosis was confirmed.

Whole exome sequencing identified a novel heterozygous interleukin receptor 1 (IL1RN) variant (c.73C>G; p. Glu25Gln) (NM_173841.3). The patient denied any related family history.

**Figure 4:**
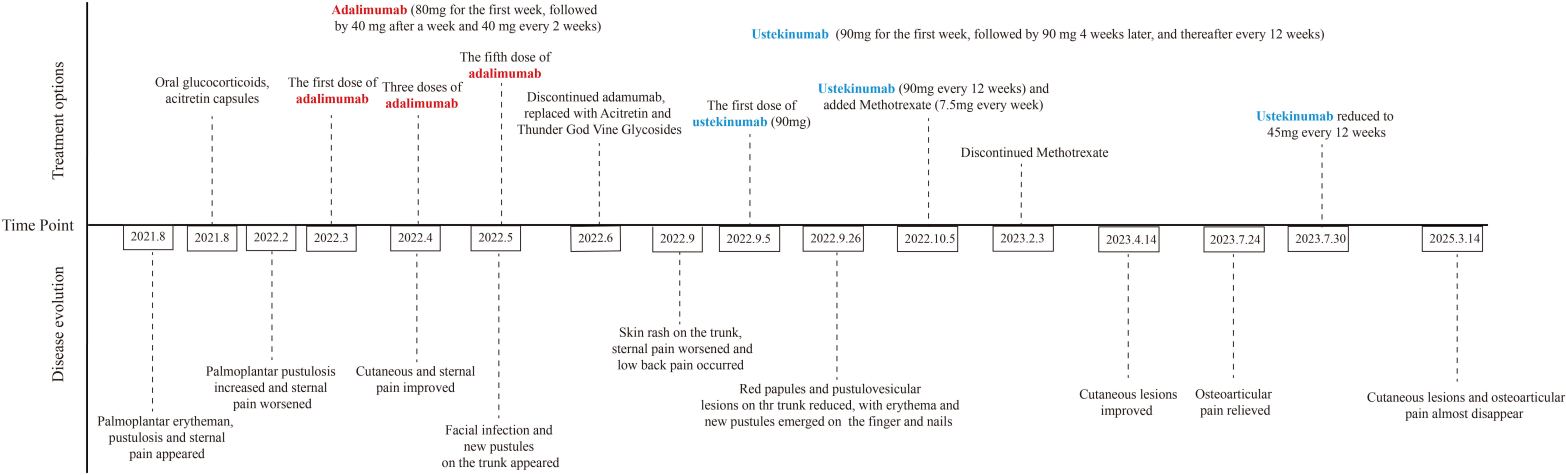
Detailed disease evolution and treatment options of the patient over time.

Following a definitive diagnosis and the patient’s refractory response to adalimumab and other immunosuppressants, ustekinumab was given. To note, due to the refractory history of the lesion and joint pain, the initial dose of ustekinumab was increased to 90 mg. The guideline for ustekinumab in adult patients with plaque psoriasis is an initial dose of 45 mg for the first dose, followed by 45mg 4 weeks after and every 12 weeks. Initially, the PPP and joint pain improved after the first injection. Therefore, we continued ustekinumab (90 mg after 4 weeks) and added doxycycline (100 mg, twice daily) and methotrexate (7.5 mg, weekly). However, pustules still recurred on the palmoplantar area after 2 doses. As a result, after 12 weeks, another two doses of 90 mg ustekinumab every 12 weeks were administered. Pustules disappeared with remaining pigmentation on the trunk and legs after 3-dose administration of ustekinumab (**Figures 1O, P**). Methotrexate was therefore discontinued. After treatment with ustekinumab with 4 doses of 90 mg, significant improvement in PPP and joint pain was observed. The dose of ustekinumab was subsequently reduced to 45mg every 12 weeks, and a regular follow-up every 3 months was conducted for nearly 3 years. The patient did not exhibit recurrence of cutaneous manifestations (**Figures 1Q–Y**). The osteoarticular pain was also relieved, with less inflammation shown in the whole-body bone scintigraphy (**Figure 2B**). The changes in disease evolution and treatment options of the patient are shown in **Figure 4**.

## Discussion

Here we present a case of refractory SAPHO syndrome with concomitant palmoplantar pustulosis (PPP) who developed exacerbations of pustules on the palms and new-onset rash on the face, trunk, and limbs after being treated with successive injections of adalimumab and acitretin. After the switched administration of ustekinumab, new-onset erythema and pustules were controlled, and the SAPHO syndrome improved.

SAPHO syndrome represents a rare clinical spectrum of inflammatory diseases characterized by osteoarticular and dermatologic manifestations (7). The most widely used diagnostic criteria were proposed by Kahn in 1994 (8), with more precise diagnostic criteria modified in 2003 based on that, both focusing on skin and osteoarticular lesions. Osteitis and hyperostosis involving the anterior chest wall and axial skeleton (including the spine and sacroiliac joints) are the core clinical manifestations of SAPHO syndrome (5). Cutaneous manifestations vary widely in severity and are composed of different acneiform and neutrophilic dermatoses. According to Hayem et al., skin involvement was documented in 68% of patients either at diagnosis or before (9). PPP constitutes the most common manifestation, occurring in up to 60% of cases, followed by severe acne in approximately 20% of patients (10). Plaque psoriasis — either isolated or associated with PPP — affects 5-20% of patients (1).

In the present case, the patient demonstrated typical clinical features: PPP; pain in the spine and sternoclavicular joints; and characteristic inflammatory changes shown on the 99mTc-methylene diphosphonate whole-body bone scintigraphy. Comprehensive evaluation ruled out infectious etiologies, bone tumors, and other spondyloarthropathies, thereby confirming the SAPHO syndrome diagnosis.

The etiology of the disease remains elusive; however, its association with Crohn’s disease, inflammatory arthritis, and psoriasis in affected individuals and their close relatives suggests shared pathophysiological mechanisms and implicates genetic predisposition (11). Of particular interest is the deficiency of IL-1 receptor antagonist (DIRA), caused by mutations in IL1RN (the gene encoding the IL-1 receptor antagonist) (12), results in dysregulation of the IL-1 pathway. Affected individuals present with neonatal onset cutaneous pustulosis, marked elevation of inflammatory markers, sterile multifocal osteitis, and periostitis (12). While classic DIRA is an autosomal recessive disorder, the patient exhibited only a heterozygous IL1RN mutation. This finding raises the possibility that heterozygous mutations may confer susceptibility to sterile inflammatory disorders like SAPHO syndrome, though further investigation is required to elucidate this potential relationship.

At present, there is no standard treatment for SAPHO syndrome based on limited experience. The main therapeutic goals focus on relieving clinical symptoms and delaying disease progression. Common treatment approaches include non-steroidal anti-inflammatory drugs (NSAIDs), disease-modifying anti-rheumatic drugs (DMARDs), corticosteroids, and antibiotics. For refractory cases, biologics have demonstrated promising efficacy. Moreover, the combination of biological agents and conventional systemic treatment has emerged as a safe and effective option (13). TNF-α inhibitors are the most widely used biologics in the treatment of SAPHO syndrome, showing benefits for both cutaneous and osteoarticular manifestations (6). Currently used TNF-α inhibitors include infliximab, adalimumab, certolizumab pegol, golimumab, and etanercept, and these agents are proved to be well tolerated (14). However, in this case, during the treatment with adalimumab, the patient developed new-onset erythema on the face, trunk, and limbs, with pre-existing PPP and pain in the sternum and spine worsened. Other dermatologic complications, such as psoriasiform lesions during treatment with TNF-α inhibitors, are reported in other cases and mainly have been associated with infliximab, etanercept (15) or adalimumab (16).

Other than TNF-α, some authors associate PPP a disease in the spectrum of psoriasis, and recently the IL-23/IL-17 inflammatory pathway has been suggested to play an important role in immune-mediated inflammatory diseases (IMID) (17), as well as in PPP (18). In addition, as is reported in other research, TNF-α inhibitors increase the secretion of interferon (IFN) by plasmacytoid dendritic cells (pDC), which leads to the activation of myeloid dendritic cells and thus stimulation of pathologic T cells (14). It was already reported that the levels of IFN-γ-secreting Th1 cells and IL-17/IL-22-secreting Th17 cells increase in patients who developed TNF-α-inhibitor-induced psoriasis (19). IL-17A and IL-17F secreted by Th17 cells have been linked to tissue neutrophil recruitment (20). Therefore, it was assumed that the IL-23/IL-17 pathway may contribute to the exacerbation of PPP driven mainly by neutrophils and uncontrolled SAPHO syndrome.

Ustekinumab is a fully humanized IgG1κ monoclonal antibody (mAb) that inhibits the p40 chain shared by IL-12 and IL-23, which is FDA approved for the treatment of plaque psoriasis and psoriatic arthritis (21). Ustekinumab is supposed to be a therapeutic approach in diseases in which neutrophil inflammation plays a role, such as in PPP. Data regarding the use of biologics targeting the IL-23/IL-17 axis in patients with PPP and SAPHO are rather limited, compared with TNF-alpha inhibitors. Anti-IL-17 antibodies are the more described, with a generally good efficacy profile, compared with anti-IL-23. Interestingly, the two case series that describe secukinumab—the most reported drug—illustrate contradictory results. While Wang et al. (22) described a satisfactory efficacy on both joints and skin symptoms in all of their four patients, no particular efficacy on the osteoarticular manifestations and only a partial efficacy on skin was reported by Wendling et al. in a series of three patients (23). The other single cases reported instead a good efficacy on bone, skin and bone manifestations (overall 11/15 skin resolution and 11/16 bone remission).

Concerning ustekinumab, few reports have been presented; thus, the efficacy of IL-23/IL-17 inhibitors is inconclusive. As was reported in 2025, response rates with ustekinumab for both osteoarticular and cutaneous symptoms were 50% (3/6) (1, 24). Firinu et al. (25) reported that subcutaneous monotherapy using ustekinumab 90 mg together with colchicine and oral steroids significantly improved skin and osteoarticular symptoms after two years of treatment, without adverse effects. Notably, carporuscio et al. demonstrated a case of SAPHO syndrome comorbid with Crohn’s disease, in which both intestinal manifestations and sternoclavicular osteitis achieved significant improvement following one year of ustekinumab therapy (26). The efficacy of ustekinumab has been described also for PPP. Four patients with refractory PPP treated with ustekinumab were reported in one clinic (27). However, in this case series, ustekinumab (90mg or 45mg subcutaneously) failed to induce a beneficial treatment response in patients with PPP. In two patients, treatment with ustekinumab was not satisfactory with fresh pustules, whereas one patient with contaminant plaque psoriasis had a good clinical response of her psoriasis, with PPP lesions only slowly improving. Ustekinumab showed a good but slow efficacy in only one patient with PPP.

The IL-23 pathway has emerged as a promising therapeutic target for SAPHO syndrome, with growing evidence supporting its clinical efficacy. Risankizumab, an anti-IL-23p19, has also been described as effective for SAPHO syndrome, with satisfactory results on all the components, including quality of life (28, 29). Ferraioli et al. (29) reported a case of SAPHO treated with risankizumab after unsuccessful therapies with methotrexate, infliximab, adalimumab, and an allergic reaction to secukinumab. Licata et al. (30) successfully treated one patient with tildrakizumab (anti-IL-23p19), improving their skin manifestations after 4 weeks. Moreover, a Japanese phase III randomized controlled trial of guselkumab (anti-IL-23p19) versus placebo in PPP, showed significant improvements in both skin manifestations and health-related quality of life (31).

Lastly, recent evidence supports the potential of JAK inhibitors for treating SAPHO syndrome (1). In particular, tofacitinib, a JAK1 and -3 inhibitor, has shown efficacy on both skin and joint involvement. Results from Li et al. demonstrated promising outcomes in a cohort of 12 patients, with 75% showing bone response and the majority achieving significant improvement in cutaneous manifestations (6 PPP, 1 acne), alongside consistent pain reduction (32). Subsequent studies found a significant improvement in both PPP and nail involvement in 13 SAPHO patients. However, osteoarticular benefits appeared limited to symptomatic pain relief without corresponding MRI changes (33). Interestingly, Ru et al. (34) reported treating a patient with coexisting Takayasu syndrome, improvement in both skin and osteoarticular symptoms, as well as a reduction in carotid artery thickness.

In conclusion, this case report provides updated evidence on the efficacy of ustekinumab in treating SAPHO syndrome. The patient experienced significant improvement in cutaneous symptoms, including clearance of skin lesions and cessation of new pustule formation, as well as notable relief of osteoarticular manifestations, supported by documented changes on bone scintigraphy. Over a three-year follow-up period, the patient maintained complete remission without any reported complications or adverse effects. In conclusion, this case highlights the potential of ustekinumab as an effective and well-tolerated therapeutic option for SAPHO syndrome, particularly in patients who have failed previous biologic treatments. Targeting the IL-12/IL-23 pathway may thus represent a promising strategy in the management of SAPHO syndrome.

## Data Availability

The original contributions presented in the study are included in the article/supplementary material. Further inquiries can be directed to the corresponding author.
